# PES1 promotes BET inhibitors resistance and cells proliferation through increasing c-Myc expression in pancreatic cancer

**DOI:** 10.1186/s13046-019-1466-7

**Published:** 2019-11-12

**Authors:** Xin Jin, Rui Fang, Ping Fan, Lipeng Zeng, Bin Zhang, Xiaoming Lu, Tao Liu

**Affiliations:** Cancer center, Union Hospital, Tongji Medical College, Huazhong University of Science and Technology, Wuhan, 430022 China

**Keywords:** PES1, BRD4, C-Myc, CDK5, Pancreatic cancer

## Abstract

**Background:**

Overexpressed PES1 promotes carcinogenesis in various types of malignant tumors. However, the biological role and clinical significance of PES1 in pancreatic cancer are still unexplored.

**Methods:**

The expression level of PES1 in pancreatic cancer cell lines and pancreatic cancer patient samples was determined using Western Blotting analysis, RT-qPCR analysis, immunohistochemical (IHC) analysis of tissue microarray, and the GEPIA web tool. MTS assay, colony formation assay, and xenograft tumor assay were used to evaluate the tumor growth ability of pancreatic cancer cells.

**Results:**

We established that the expression of PES1 was abnormally increased in pancreatic cancer tissues and led to poor prognosis of pancreatic cancer patients. We also found that PES1 was responsible for promoting cell growth and contributed to bromodomain and cancer cell resistance to extra-terminal (BET) inhibitors in pancreatic cancer. Furthermore, we showed that PES1 interacted with BRD4 to enhance c-Myc expression, which is the primary cause of cancer cell resistance to BET inhibitors in pancreatic cancer. Finally, CDK5 inhibitors were proven to destabilize PES1 and overcome cancer cell resistance to BET inhibitors in pancreatic cancer cells.

**Conclusions:**

We have shown that PES1 could be one of the promoting factors of tumor growth and a prognosis-related protein of pancreatic cancer. Targeting PES1 with CDK5 inhibitors might help overcome cancer cell resistance to BET inhibitors in pancreatic cancer cells.

## Background

Pancreatic cancer is one of the most lethal digestive system malignant tumors in the world [1]. Surgical resection is the primary therapeutic strategy for pancreatic cancer, but most patients lose the chance to undergo curative surgical treatment because of late-stage diagnoses [2]. Other corrective measures, such as chemotherapy and radiotherapy, have shown limited effects on prolonging the survival time of pancreatic cancer patients [3, 4]. With all these limitations to the current procedure, it is, therefore, of extraordinary importance to explore new therapeutic methods for pancreatic cancer.

Molecular target therapy is a treatment that seems promising for numerous cancer types [5]. Genome sequencing of pancreatic cancer patient specimens has uncovered multiple types of mutations, such as Kras, TP53, CDKN2A, and aberrant activation or inactivation of cellular signaling pathways, including the MAPK and PI3K/AKT pathways, which are potential candidates for molecular target therapy [6]. Several small molecular chemicals have been designed to inhibit tumor proliferation [7, 8]. Among these chemicals are BET inhibitors that have been revealed to show anti-tumor effects in pancreatic cancer, with some BET inhibitors, such as JQ1, having been tested in clinical trials [9], although cancer cell resistance to BET inhibitors hinders the use of molecules clinically [10]. Bromodomain-containing protein 4 (BRD4) belongs to the bromodomain and extra-terminal (BET) family, which acts as a co-activator of transcription factors to increase the expression of various oncogenic genes, such as Myc [11]. BRD4 helps in cancer cell resistance to BET inhibitors. Consequently, establishing the mechanism of cancer cell resistance to BET inhibitors might improve the outcome of pancreatic cancer patients.

Pescadillo ribosomal biogenesis factor 1 (PES1), an encoder of a nuclear protein that contains the C-terminal interaction domain of breast cancer-associated gene 1 (BRCA1) [12], is overexpressed in various kinds of solid tumors, such as breast cancer [13], colon cancer [14], liver cancer, ovarian cancer [15], and thyroid cancer [16]. The aberrant expression of PES1 is involved in ER balance [13], cell cycle regulation [14], and PI3K/AKT pathway activation [17]. Highly expressed PES1 results in cancer cell proliferation and malignant transformation and the poor prognosis in multiple types of cancer [18, 19]. The specific role of PES1 in pancreatic cancer is, however, still not clear.

Here, we examine the expression level of PES1 in pancreatic cancer patient specimens to determine its clinical significance and explore its biological role in pancreatic cancer cells. Next, we evaluate the effect of PES1 on the sensitivity of small anti-tumor molecules and determine if PES1 contributes to cancer cell resistance to BET inhibitors by upregulating c-Myc expression. Finally, we assess CDK5 inhibitors’ ability to destabilize the PES1 protein and overcome cancer cell resistance to BET inhibitors in pancreatic cancer.

## Method and material

### Plasmids and reagents

Flag-PES1 was cloned into the CMV-MCS-3xFlag-SV40-neomycine vector by GENECHEM (Shanghai, China). Flag-CDK5 was cloned into the pcDNA3.0 vector reported previously [20]. KOD-Plus- Mutagenesis Kit (Cat #SMK-101B, TOYOBO) was used to generate Flag-PES1 K537R/K540R, S424A, S424D mutants respectively.

Antibodies used were: PES1 (Abcam, ab72539, working dilution 1:200), GAPDH (Abcam, ab8245, working dilution 1:5000), CDK5 (Cell Signaling Technology, 2506, working dilution 1:1000), Myc (Abcam, ab32072, working dilution 1:1000), BRD4 (Cell Signaling Technology, 13,440, working dilution 1:1000).

Reagents used were: Palbociclib (PD0332991) (Cat. No. S1579), JQ1 (Cat. No. S7110), MK2206 (Cat. No. S1078), GSK126 (Cat. No. S7061), Dinaciclib (SCH727965) (Cat. No. S2768), Everolimus (RAD001) (Cat. No. S1120), MK1775 (Cat. No. S1525), p38 MAPK inhibitor (SB203580) (Cat. No. S1076), Olaparib (Cat. No. S1060), Trichostatin A (TSA) (Cat. No. S1045), Roscovitine (Cat. No. S1153), PD0325901 (Cat. No. S1036) and MG 132 (Cat. No. S2619) were purchased from Selleckchem.

### Xenografts transplantation model in nude mice

The BALB/c-nude mice (4–5 weeks of age, 18-20 g) were purchased from Vitalriver (Beijing, China). PANC-1 and BxPC-3 cells were infected with shControl or shPES1. After puromycin selection for 72 h, cells (5 × 10^6^/each mouse) were subcutaneously inoculated in the left back side of mice. The length and width of xenografts was measured using vernier caliper and their volumes were figured up by the formula (L × W^2^)/2. All mice were euthanized after subcutaneous implantation 21 days and then all xenografts were excised to weight. Secondly, PANC-1 cells were infected with shControl or shPES1. After puromycin selection for 72 h, cells (5 × 10^6^/each mouse) were subcutaneously inoculated in the left back side of mice. After the tumor volume was approximately 100 mm^3^, mice were treated with DMSO or JQ1 (50 mg/kg i.p. twice weekly). All mice were euthanized after subcutaneous implantation 30 days and then all xenografts were excised to weight. Thirdly, PANC-1 cells (5 × 10^6^/each mouse) were subcutaneously inoculated in the left back side of mice. After the tumor volume was approximately 100 mm^3^, mice were treated with DMSO, JQ1 (50 mg per kg bodyweight (intraperitoneal injection)), Dinaciclib (40 mg/kg i.p. twice weekly) [21] and JQ1 plus Dinaciclib respectively. All mice were euthanized after subcutaneous implantation 30 days and then all xenografts were excised to weight. All animal experiment procedures were approved by the Ethics Committee of Tongji Medical College, Huazhong University of Science and Technology.

### Tissue microarray and immunohistochemistry (IHC)

IHC analysis was performed to research the role of PES1 in pancreatic cancer, including the change of PES1 expression in PDAC, the relationship between PES1 and c-Myc by using the tissue microarray (Cat No. XT14–029, Outdo Biobank, Shanghai, China), the variation of Cleaved-caspase-3 expression after knocking down PES1 from the xenografts. Antibody used: PES1 (Abcam, ab72539, working dilution 1:200) and c-Myc (Santa Cruz Biotechnology, 5605P, working dilution 1:100). Besides, the xenografts from the nude mice were embedded by paraffin and made tissue sections to carry out Cleaved-caspase-3 (Proteintech, 25,546–1-AP, working dilution 1:1000) immunostaining. The IHC score was calculated as the product of the staining intensity score and the proportion of positive tumor cells. The staining intensity was graded according to following criteria: 1 = weak staining at 100× magnification but little or no staining at 40× magnification; 2 = medium staining at 40× magnification; 3 = strong staining at 40× magnification. The immunostaining intensity was scored and the proportion of positive tumor cells was determined according to the proportion of positive cells in all cells in each case by two experienced pathologists independently who didn’t know the detailed information.

### Immunoprecipitation and Western blot analysis

The ethics of using human tissue (11 pairs of matched pancreatic cancer/adjacent noncancerous tissues) was approved by the local ethics committee (Tongji Medical College, China), and written informed consent was obtained from patients prior to surgery exactly as described previously [10]. For immunoprecipitation, cells were harvested and resuspended in 1 ml of RIPA buffer for 15 min. Cell lysate was centrifuged for 15 min at 13200 r.p.m. at 4 °C. The supernatant incubated with Pierce Protein G Agarose and primary antibody or IgG in the cold room overnight. The beads were washed five times with IP buffer, resuspended with sample loading buffer and heated at 100 °C for 5 min. The supernatant was used for further western blotting analysis. The whole cell lysates of pancreatic cancer cells were obtained after adding with 1x RIPA buffer added 1 mM PMSF (Phenylmethanesulfonyl fluoride) immediately before use. The WCL balanced by BCA method. Equal amount of WCL was separated by the SDS-PAGE gel and transferred to the PVDF membrane. Then, the membrane containing target protein was incubated with the primary antibody at 4 °C for more than 8 h. Next, the second antibody was used for the incubation of membrane for 1 h at room temperature. The protein was detected by the Chemiluminescent Western Blot Detection Kit (Cat No. 32209, Thermo Fisher Scientific, USA).

### Cell proliferation assay

For MTS cell growth assay, equal number of pancreatic cancer cells were plated into the 96 well plate and added MTS reagent according to the manufacture’s protocol (Cat No.Ab197010, Abcam). The absorbance at 490 nm was used for the evaluation of cell proliferation rate.

For CCK 8 assay, 1000 cells were placed in each well of the 96-well plate, and fresh medium containing 10ul CCK 8 reagent (Cat No. K1018, APExBIO) was replaced at the same time point of the first, second, third and fourth days, respectively, and incubated at 37° and 5% CO2 for 1 h. Absorbance value was measured at the wavelength of 450 nm.

For BrdU cell proliferation assay, 1000 cells were placed in each well of the 96-well plate. The adherent cells were washed with PBS three times, and the proliferation of the cells was detected with the BrdU cell proliferation kit according to the manufacturer’s instructions (Cat No. 6813, Cell Signaling Technology). Absorbance value was measured at the wavelength of 450 nm.

### In vitro CDK5 kinase assay

Wild-type Flag-PES1 and mutant Flag-PES1 (S424A) proteins were translated in vitro following the manufacture protocol of TNT® Quick coupled Translation System Technical (Promega, Cat No. TM045) as described previously [22]. These proteins were purified with Pierce Protein G Agarose and primary antibody (Flag-tag antibody, Cat No. A5712, Bimake) in the cold room overnight. Then, the purified proteins were added into kinase assay buffer (Cat No.ab189135, Abcam), and incubated with activated CDK5/p25 (Cat No. ab60761, Abcam) and 50 μM ATP-γ-S (Cat No. ab138911, Abcam) at 30 °C for 45 min. 2.5 mM PNBM/5% DMSO were added to the sample at the room temperature for 1 h. The phosphorylated protein was detected by an anti-thiophosphate ester antibody (Cat No. ab92570, Abcam) [23].

### Detection of apoptosis using flow cytometry

The adherent cells were digested into a single cell suspension by trypsin without EDTA and apoptosis was detected using Annexin v-fitc apoptosis assay kit (Cat No BA1150, EnoGene). Then, cell suspension samples were analyzed on BD FACSCanto II (BD Biosciences, USA) with data analyzed using FlowJo software.

### Statistical analysis

All data are presented as the means ± SDs. Comparisons between groups were calculated by one way or two ways ANOVA using GraphPad Prism 5 software. *P* < 0.05 was considered statistically significant.

Other methods were provided in the Supplementary information.

## Results

### Overexpressed PES1 might be one of the prognostic biomarkers for pancreatic cancer

**Fig. 1:**
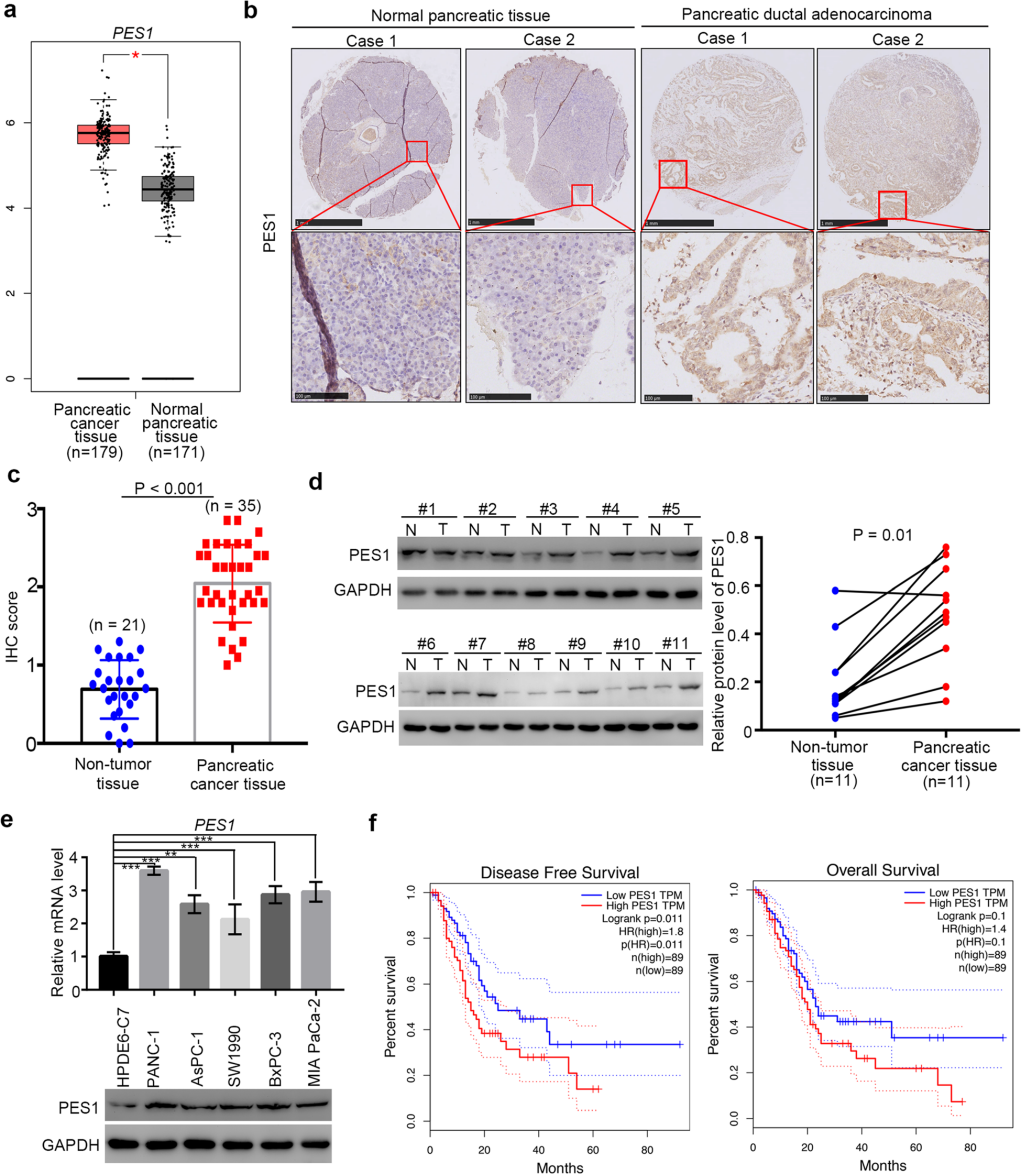
Overexpressed PES1 might be one of the prognostic biomarkers for pancreatic cancer. **a**, the mRNA expression level analysis of PES1 in pancreatic cancer patient specimens (*n* = 179) and pancreatic non-tumor tissues (*n* = 171) by using the GEPIA web tool (http://gepia.cancer-pku.cn/). *, *P* < 0.05. **b**, typical IHC image of PES1 in pancreatic tissue microarray (pancreatic non-tumor tissue *n* = 21, pancreatic cancer *n* = 35). **c**, IHC scores of PES1 in pancreatic tissue microarray (pancreatic non-tumor tissue n = 21, pancreatic cancer n = 35), *P* < 0.001. **d**, the expression level of PES1 analyzed by Western Blotting from 11 pair of pancreatic cancer patient specimens and adjacent non-tumor tissues. *P* = 0.01. **e**, HPDE6-C7, PANC-1, AsPC-1,SW1990, BxPC-3 and MIA PaCa-2 cells were harvested for Western Blotting and RT-qPCR analysis. Data presented as Means ± SD (n = 3). **, *P* < 0.01; ***, P < 0.001. **f**, the disease free survival time and overall survival time of pancreatic cancer patients with different expression level of PES1 was determined by GEPIA, *P* values as indicated

Multiple studies have mentioned that PES1 is overexpressed in various types of cancer tissues, including breast cancer [13], liver cancer [19], and gastric cancer [24], and that it participates in tumorigenesis by modulating cancer cell proliferation, apoptosis, and metabolism processes [18, 19]. However, the role of PES1 in pancreatic cancer is poorly understood. Notably, as PES1 is up-regulated by CD44, c-Jun, and BRD4 in liver or colon cancer cells [14, 19]. Besides, it has been reported that CD44, c-Jun, and BRD4 promote pancreatic cancer cell proliferation and metastasis and induce chemotherapy resistance [25–28]. As a consequence, we pondered whether PES1 was overexpressed in pancreatic cancer. After analyzing patient datasets with the GEPIA web tools [29], we demonstrated that pancreatic cancer tissues exhibited more profound PES1 expression than non-tumor tissues (Fig. 1a).

Also, the tissue microarray of pancreatic cancer, containing 21 cases of non-tumor pancreatic tissue samples and 35 cases of pancreatic cancer tissue specimens, was subjected to immunohistochemical (IHC) analysis to evaluate the expression of PES1 (Fig. 1b and c). Similarly to results obtained with the GEPIA web tools, PES1 was up-regulated significantly in pancreatic cancer tissues (Fig. 1b and c). Moreover, Western Blotting analysis of 11 pairs of pancreatic cancer patients with adjacent non-tumor pancreatic tissues revealed that PES1 was highly present in pancreatic cancer tissues (Fig. 1d).

Furthermore, the expression levels of PES1 in human healthy pancreatic ductal epithelial cells and human pancreatic cancer cells are shown in Fig. 1e. We revealed that PES1 expression in pancreatic cancer cells was higher than that in healthy pancreatic ductal epithelial cells (HDPE6-C7). These assessments suggest that PES1 is aberrantly expressed in pancreatic cancer.

We also found that high expression levels of PES1 resulted in shorter survival times in pancreatic cancer patient specimens (Fig. 1f). Thus, our data indicate that overexpressed PES1 might be a prognostic biomarker for pancreatic cancer.

### PES1 enhances pancreatic cancer cell growth in vitro and in vivo

**Fig. 2:**
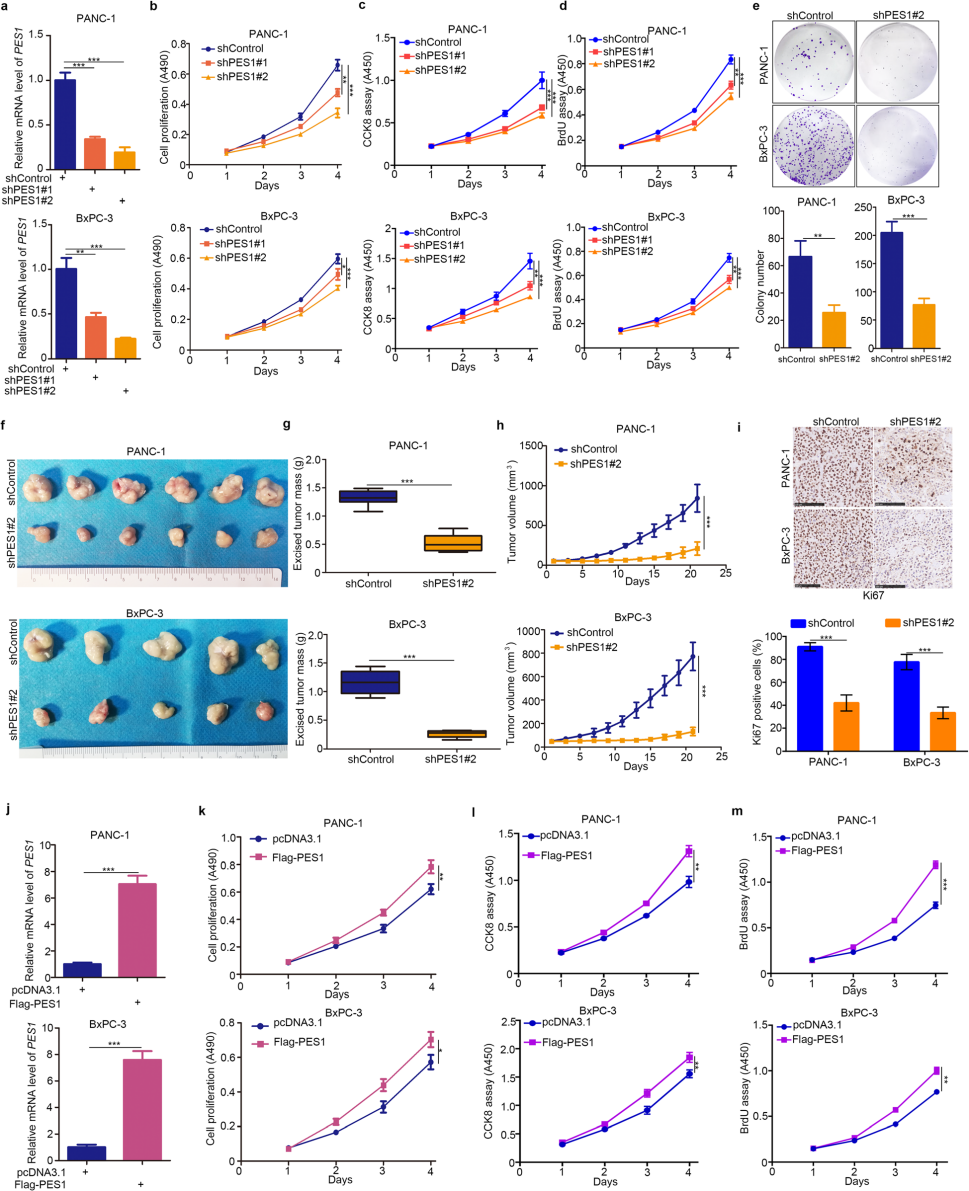
PES1 enhances pancreatic cancer cell growth in vitro and in vivo**. a-e,** PANC-1 and BxPC-3 were infected with indicated constructs. After 72 h, cells were harvested for RT-qPCR analysis (a), MTS assay (b), CCK8 assay (c), BrdU assay (d) and colony formation assay (e). Data presented as Means ± SD (*n* = 3). *, *P* < 0.01; **, *P* < 0.01; ***, *P* < 0.001. **f-i**, pancreatic cancer cell lines (PANC-1 and BxPC-3) were transfected with indicated constructs. 72 h post-transfection, cells were injected subcutaneously into the nude mice for xenografts assay for 21 days. The image of xenografts was shown in (f), the tumor mass and volume of xenografts was determined in (g) and (h). The Ki-67 staining was shown in panel i. Data presented as Means ± SD (*n* = 6 for PANC-1 and *n* = 5 for BxPC-3). ***, *P* < 0.001. **j**-**m**, PANC-1 and BxPC-3 cells were transfected with indicated plasmids. After 24 h, cells were harvested for RT-qPCR analysis (**j**), MTS assay (**k**), CCK8 assay (**l**) and BrdU assay (**m**). Data was shown in the form of Means ± SD (*n* = 3). *, P < 0.05; **, *P* < 0.01; ***, *P* < 0.001

Given PES1’s clinical importance to pancreatic cancer patients (Fig. 1), we considered whether PES1 had any effect on the biological behavior of pancreatic cancer cells. Firstly, we suppressed the expression levels of PES1 in pancreatic cancer cells using specific short hairpin RNA (shRNA) (Fig. 2a). MTS, CCK8, BrdU cell proliferation assay, and colony formation assay were used to determine cell growth ability after knocking down PES1 in pancreatic cancer cells (Fig. 2b-2e). Our data demonstrate that the inhibition of PES1 markedly slowed down pancreatic cancer proliferation in vitro.

A xenograft tumor assay was next employed to evaluate cancer cell proliferation ability in vivo (Fig. 2f). We observed that the knockdown of PES1 by shRNA profoundly blocked pancreatic cancer cell growth and decreased Ki-67 positive cells in the tumor (Fig. 2f-2i). On the contrary, up-regulating the levels of PES1 by ectopically transfecting corresponding plasmids enhanced the proliferation ability of pancreatic cancer cells (Fig. 2j and m). Therefore, our data suggest that PES1 acted as a growth-promoting protein for pancreatic cancer cells in vivo and in vitro.

### Knocking down PES1 increases pancreatic cancer cell sensitivity to BET inhibitors

**Fig. 3:**
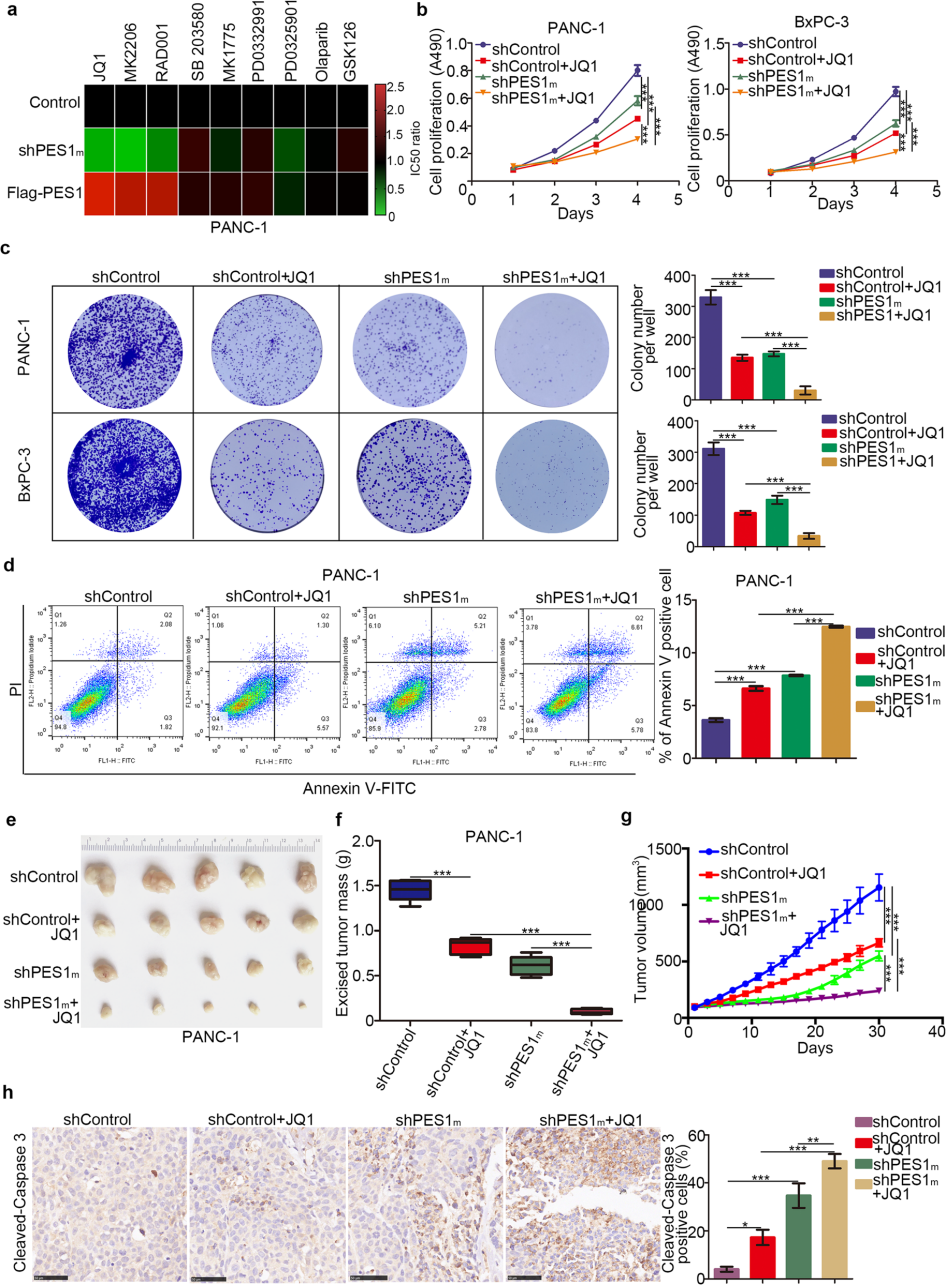
Knocking down PES1 increases pancreatic cancer cell sensitivity to BET inhibitors. **a,** PANC-1 cells were transfected with indicated plasmids for 72 h. Cells were treated the different types of small inhibitors for 48 h, the IC50 values was analyzed and IC50 ratio of Control vs shPES1m and Control vs Flag-PES1 were determined and shown in heatmap. shPES1m indicated that mixed shRNA (shPES1#1 and shPES1#2). **b** and **b**, pancreatic cancer cells were infected with indicated plasmids for 72 h. Then, cells were treated with or without JQ1 (5uM) for MTS assay and colony formation assay. Data presented as Means ± SD (*n* = 3). **, P < 0.01; ***, P < 0.001. **d,** PANC-1 cells were infected with indicated plasmids for 72 h. Then, cells were treated with or without JQ1 (5uM) for 48 h. Cells were subjected to Annexin-V/Propidium Iodide (PI) assay. Data presented as Means ± SD (n = 3). ***, P < 0.001. **e-h**, PANC-1 cells were infected with indicated shRNA for 72 h. Cell were subcutaneously injected into the nude mice. These mice were treated with or without JQ1 for 27 days. These tumors were harvested for photograph (**e**), weight (**f**), tumor growth curve mapping and caspase 3 analysis by IHC (**h**). Data presented as Means ± SD (n = 5 for e, f and g, *n* = 3 for h). *, P < 0.05; **, P < 0.01; ***, P < 0.001

To explore the effect of PES1 further, we evaluated the sensitivity of small molecular drugs after knockdown or overexpression of PES1 in PANC-1 cells (Fig. 3a). Briefly, PES1 was knocked down or overexpressed in PANC-1 following treatment with a series of small molecules (JQ1, MK2206, RAD001, SB203580, MK1775, PD0332991, PD0325901, Olaparib, GSK126). The IC50 of every group was measured to determine the drug sensitivity of these molecules. The IC50 values of these inhibitors in the PES1 knockdown/overexpressed group were normalized to the IC50 value in the control group and are shown in the form of a Heatmap in Fig. 3a. Our results indicated that PANC-1 cells were sensitive to BET inhibitors, AKT inhibitors, and mTOR pathway inhibitors after the knockdown of PES1 (Fig. 3a). In contrast, increasing the levels of PES1 resulted in PANC-1 cells’ resistance to BET inhibitors, AKT inhibitors, and mTOR pathway inhibitors (Fig. 3a).

Because PES1 is reported to activate PI3K/AKT signaling in liver cancer cells [17], which is consistent with our findings in Fig. 3a, we focused on the BET inhibitors-related pathway in pancreatic cancer. To verify PES1’s role in the sensitivity of pancreatic cancer cells to BET inhibitors, we established stable PES1 knockdown pancreatic cancer cells using mixed shPES1 (shPES1m). MTS assays and colony formation assay demonstrated that BET inhibitors suppressed cancer cell growth in the PES1 knockdown group more than they did in the control group (Fig. 3b and c). On the other hand, the repression of PES1 led to more cancer cell apoptosis after treatment with BET inhibitors (Fig. 3d).

In vivo studies also confirmed that the growth of tumors was slower (Fig. 3e-3g), and there were more apoptotic cells in the group when knocking down PES1 and using BET inhibitors simultaneously compared to the control group employing BET inhibitors only. Additionally, we revealed that recusing PES1 expression in the PES1 knockdown group caused resistance to JQ1 in PANC-1 cells (Additional file 1 Figure S1a-1c). Meanwhile, JQ1 showed a pretty good inhibitory effect against PANC-1 by suppressing IL-6, CCL2, and GM-CSF [9]. Our data demonstrated further that JQ1 triggered more decrease in IL-6, CCL2, and GM-CSF levels in the PES1 knockdown group than in the control group, suggesting that PES1 repression enhanced the function of JQ1 in PANC-1 cells (Additional file 1 Figure S1f). Our data, therefore, indicate that PES1 silencing mediated the sensitivity of PANC-1 cells to BET inhibitors in pancreatic cancer.

### PES1 up-regulates c-Myc in pancreatic cancer cells

**Fig. 4:**
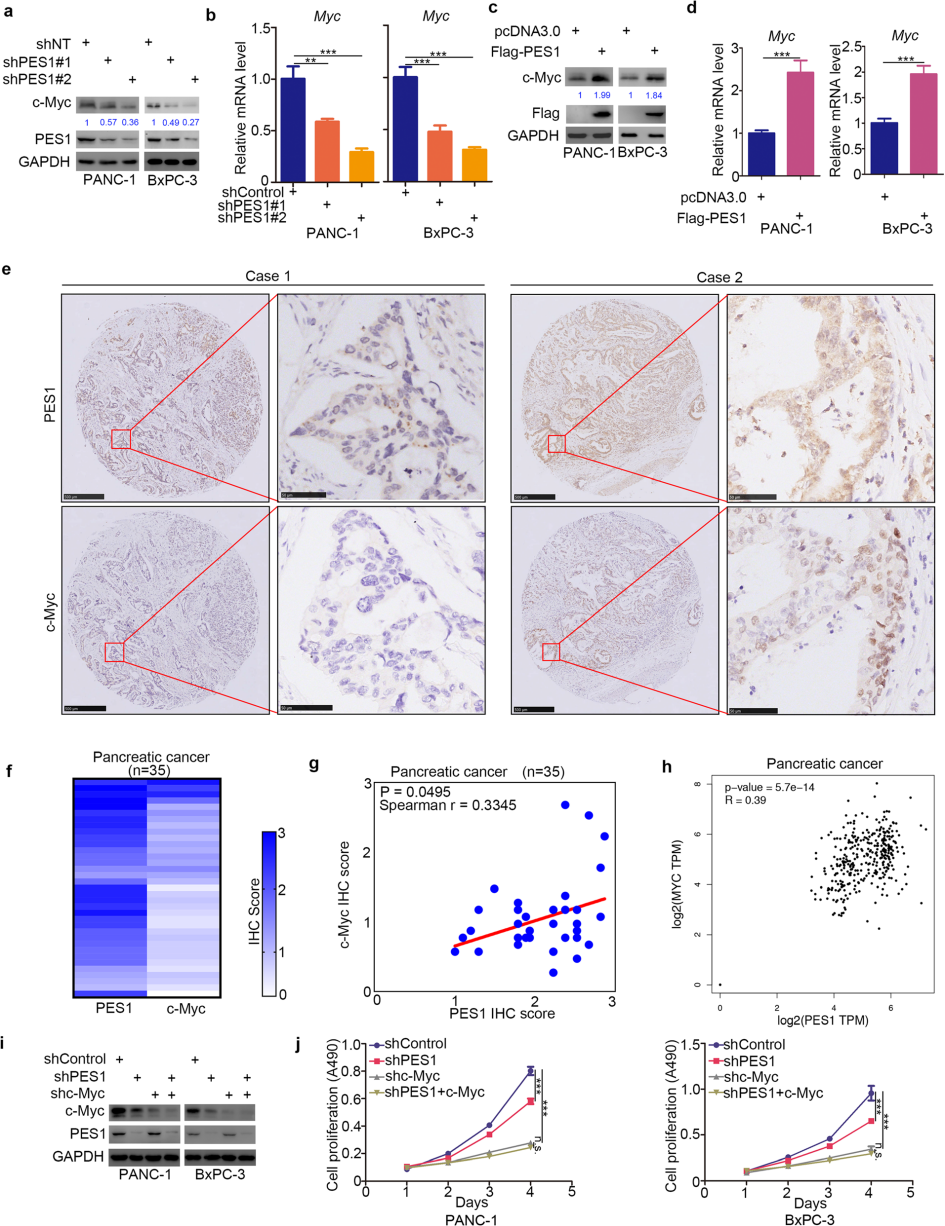
PES1 up-regulates c-Myc in pancreatic cancer cells. **a** and **b,** pancreatic cancer cells (PANC-1 and BxPC-3) were infected with indicated shRNA. After 72 h, cells were harvested for Western Blotting analysis (a) and RT-qPCR analysis (b). Data shown is in the form of Means ± SD (n = 3). **, P < 0.01; ***, P < 0.001. **c** and **d**, PANC-1 and BxPC-3 cells were transfected with indicated plasmids. After 24 h, cells were harvested for Western Blotting analysis (**c**) and RT-qPCR analysis (**d**). Data shown is in the form of Means ± SD (n = 3). ***, P < 0.001. **e-g**, the tissue microarray of pancreatic cancer (n = 35) was stained with PES1 and c-Myc respectively. The typical image of PES1 and c-Myc was shown in (**e**), the IHC scores of PES1 and c-Myc was shown in (**f**) and the correlation of these two proteins was shown in (**g**) (Spearman r = 0.3345, *P* = 0.0495). **h**, the mRNA expression level of PES1 and Myc were presented in (h) by using the GEPIA web tools (Spearman r = 0.39, P values as indicated). **i** and **j**, pancreatic cancer cell lines were infected with indicated plasmids. 72 h post-infection, cells were harvested for Western Blotting analysis and MTS assay (*n* = 3). Data presented as Means ± SD. N.s., not significant; ***, P < 0.001

Reports show that multiple factors contribute to cancer cell resistance to BET inhibitors [30–33], with c-Myc one of the factors that play a critical role in the resistance to BET inhibitors in pancreatic cancer cells [10, 33, 34]. In that regard, we assessed PES1’s ability to regulate the expression of c-Myc in pancreatic cancer cells. We first inhibited PES1 using two independent shRNAs and evaluated any potential change in the expression of c-Myc (Fig. 4a and b). We observed that the mRNA and protein levels of c-Myc decreased after the knockdown of PES1 in pancreatic cancer cells (Fig. 4a and b). In contrast, c-Myc was up-regulated in BxPC-3 and PANC-1 cells after PES1 overexpression (Fig. 4c and d).

We next investigated the relationship between PES1 and c-Myc in pancreatic cancer patient specimens via tissue microarray (Fig. 4e and f) and found that PES1 protein expression levels correlated positively with c-Myc expression levels (Spearman correlation coefficient r = 0.3345, *P* = 0.0495) (Fig. 4g). PES1 mRNA expression levels also correlated positively with Myc in pancreatic cancer patients (Fig. 4h).

We also pondered whether c-Myc is the key to PES1-induced cancer cell proliferation in pancreatic cancer. Our findings revealed that the co-knockdown of c-Myc and PES1 did not inhibit cell growth further, compared to the knockdown of c-Myc alone in pancreatic cancer cells (Fig. 4i and j). Collectively, our results indicate that c-Myc is regulated by PES1 and acts as a significant mediator of PES1-induced cell proliferation in pancreatic cancer cells.

### PES1 cooperates with BRD4 to regulate Myc expression in pancreatic cancer

**Fig. 5:**
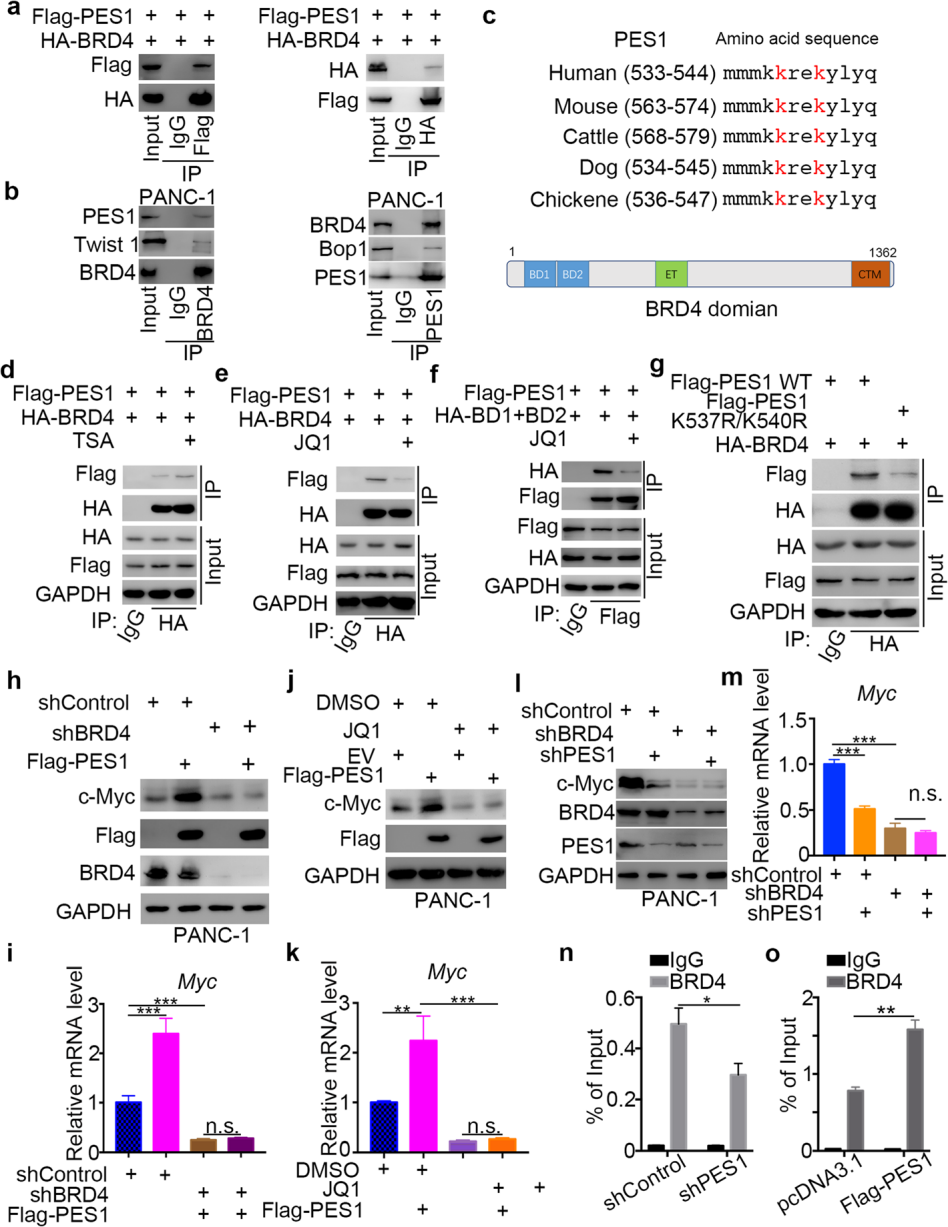
PES1 cooperates with BRD4 to regulate Myc expression in pancreatic cancer. **a**, 293 T cells were transfected with indicated plasmids. After 24 h, the whole cell lysates were harvested for Western Blotting analysis. **b**, the whole cell lysates of PANC-1 cells were harvested for Western Blotting analysis. **c**, schematic diagram depicting that a potential acetylated lysine consensus motif containing in the PES1 and the domain of BRD4. **d**, PANC-1 cells were transfected with indicated plasmids for 24 h. After other 24 h TSA (3 nM) treatment, cells were harvested for Western Blotting analysis. **e**, PANC-1 cells were transfected with indicated plasmids for 24 h. After other 24 h JQ1 (2uM) treatment, cells were harvested for Western Blotting analysis. **f**, PANC-1 cells were transfected with indicated plasmids for 24 h. After other 24 h JQ1 (2uM) treatment, cells were harvested for Western Blotting analysis. **g**, the whole cell lysates were harvested for Western Blotting analysis after transfection with indicated plasmids. **h** and **i**, PANC-1 cells were harvested for Western Blotting analysis and RT-qPCR analysis after transfection with indicated plasmids for 72 h. Data is the form of Means ± SD (*n* = 3). N.s., not significant; ***, *P* < 0.001. **j** and **k**, PANC-1 cells were transfected with empty vector (EV) or Flag-PES1 for 24 h. After other 24 JQ1 (3uM) treatment, cells were harvested for Western Blotting analysis and RT-qPCR analysis. Data is the form of Means ± SD (n = 3). N.s., not significant; **, *P* < 0.01; ***, P < 0.001. **l** and **m**, PANC-1 cells were infected with indicates shRNAs. 72 h post-infection, cells were harvested for Western Blotting analysis and RT-qPCR analysis. Data is the form of Means ± SD (n = 3). N.s., not significant; ***, P < 0.001. **n**, BRD4 ChIP-qPCR of Myc in PANC-1 cells after infected with indicated shRNA for 72 h. Data is in the form of Means ± SD (n = 3). *, *P* < 0.05. **o**, schematic diagram depicting that PES1 interacted with BRD4 to increase the expression of Myc

c-Myc is regulated transcriptionally by PES1 in pancreatic cancer cells, but the underlying mechanism remains unclear. PES1 also contributes to cancer cell resistance to BET inhibitors in pancreatic cancer. The specific role of BET inhibitors is to block the function of bromodomain-containing proteins, mainly BRD4, which modulates c-Myc. Given the connections between c-Myc, PES1, and BET inhibitors, we explored the possibility of an interaction between PES1 and BRD4 in pancreatic cancer cells. The co-immunoprecipitation assay demonstrated that PES1 binds with BRD4 exogenously (Fig. 5a) in 293 T cells and endogenously in PANC-1 cells (Twist 1 [35] and Bop1 [36] as internal reference of co-IP assay for BRD4 and PES1 respectively) (Fig. 5b). We then analyzed the amino acid sequence of PES1 and observed that PES1 contained conserved lysine sequences in different species, which could be acetylated and recognized by the bromodomain of BRD4 (BD1 and BD2) (Fig. 5c).

To verify whether the interaction between PES1 and BRD4 was acetylation-dependent, we treated cells with HDAC inhibitors (Trichostatin A, TSA) to increase the acetylation of PES1 in PANC-1 cells. Our results indicated that TSA treatment increased the binding between PES1 and BRD4 (Fig. 5d). Moreover, we found that BET inhibitors (JQ1) impeded the interaction between PES1 and BRD4 or the bromodomain of BRD4 (BD1 and BD2) in pancreatic cancer cells (Fig. 5), which suggested that PES1 might bind to the bromodomain of BRD4.

Next, we constructed the mutagenesis plasmids of PES1, PES1 K537R/560R, to mimic the de-acetylation status of PES1 and found that de-acetylation of PES1 led to decreased binding to BRD4 compared with normal PES1 in PANC-1 cells (Fig. 5g). Together, our results suggest that PES1 interacted with BRD4 in an acetylation-dependent manner.

We investigated whether c-Myc is regulated by PES1/BRD4 signaling and found that the overexpression of PES1 increased c-Myc expression, and this process was attenuated when BRD4 was knocked down in PANC-1 cells (Fig. 5h and i). Similarly, BET inhibitors (JQ1) blocked the increase in c-Myc induced by PES1 in PANC-1 cells (Fig. 5j and k).

Moreover, when we knocked down PES1 and BRD4 alone or both of them simultaneously in PANC-1 cells using shRNAs, we showed that the co-knockdown of PES1 and BRD4 did not decrease the expression of c-Myc further, compared with the knockdown of BRD4 alone (Fig. 5l and m). Furthermore, we found that the inhibition of PES1 decreased its binding to BRD4, hence, lessening the promotion of Myc (Fig. 5n), but the overexpression of PES1 enhanced this binding (Fig. 5o). Collectively, our data suggest that PES1 interacted with BRD4 and initiated the transcription of Myc in pancreatic cancer cells.

### CDK5 inhibitors destabilize PES1 and increase the sensitivity of pancreatic cancer cells to BET inhibitors

**Fig. 6:**
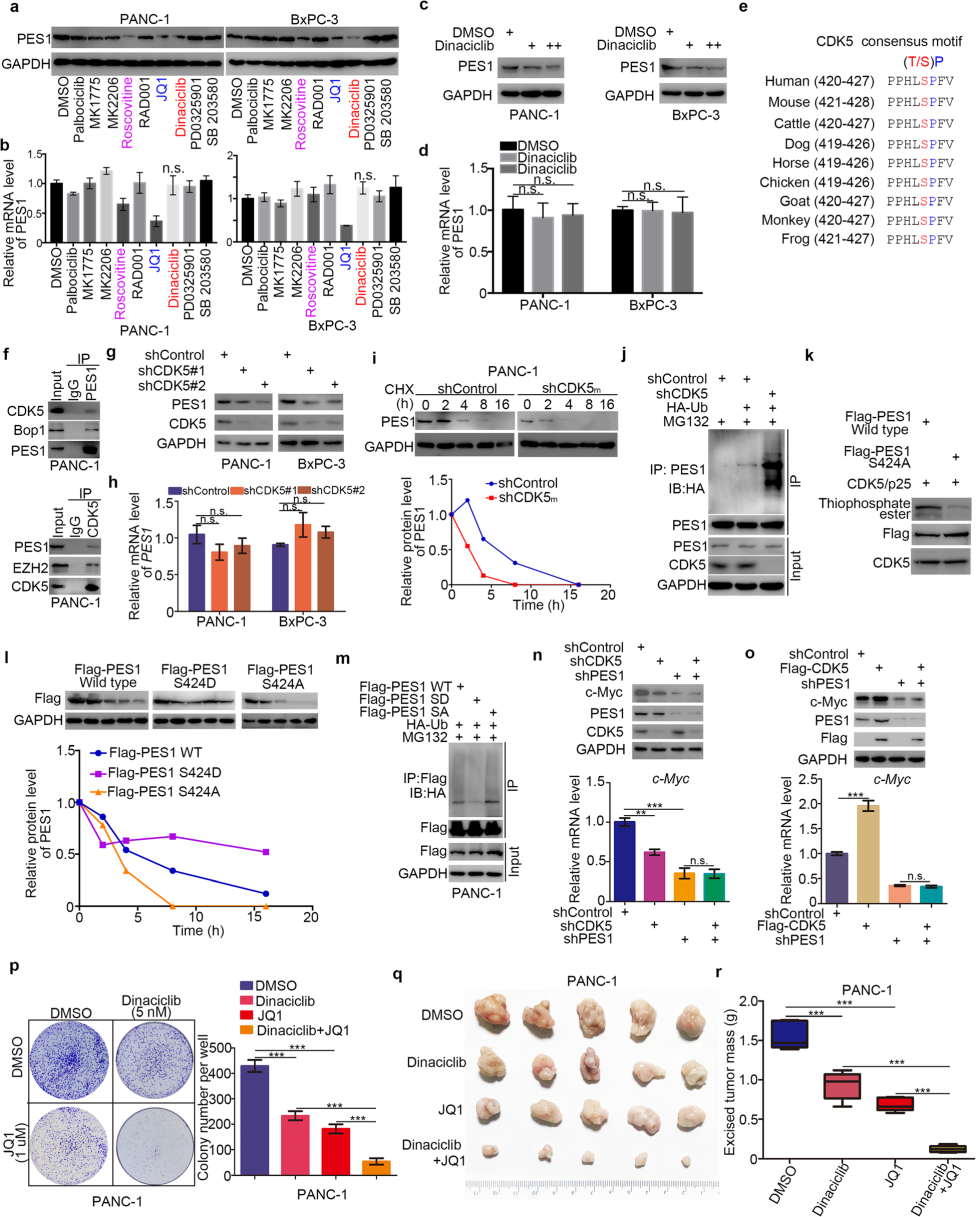
CDK5 inhibitors destabilize PES1 and increase the sensitivity of pancreatic cancer cells to BET inhibitors. **a** and **b,** PANC-1 and BxPC-3 cells were treated with DMSO, Palbociclib (5 uM), MK2206 (5 uM), MK1775 (0.5 uM), Roscovitine (1 uM), SB203580 (0.5 uM), RAD001 (5 nM), Dinaciclib (5 nM), PD0325901 (1 nM), JQ1 (1 uM) for 48 h. Cells were harvested for Western Blotting analysis (**a**) and RT-qPCR analysis (**b**). Data presented as Means ± SD (n = 3). N.s., not significant. **c** and **d,** PANC-1 and BxPC-3 cells treated with different dose of Dinaciclib for 48 h. Cells were harvested for Western Blotting analysis (**c**) and RT-qPCR analysis (**d**). Data presented as Means ± SD (*n* = 3). N.s., not significant. **e,** schematic diagram depicting that the CDK5 phosphorylation consensus motif of PES1. **f**, the whole cell lysates of PANC-1 were harvested for Western Blotting analysis. **g** and **h**, pancreatic cancer cell lines (PANC-1 and BxPC-3) were infected with indicated construct. After 72 h, were harvested for Western Blotting analysis and Rt-qPCR analysis. Data presented as Means ± SD (*n* = 3). N.s., not significant. **i**, PANC-1 cells were transfected with indicated plasmids. After 24 h, cells were treated with Cycloheximide (CHX) and cells were collected for Western Blotting analysis at different time points. **j**, PANC-1 cells were transfected with indicated plasmids. After 72 h, cells were harvested for Western Blotting analysis. **k**, Wild-type Flag-PES1 and mutant Flag-PES1 (S424A) proteins were translated in vitro. These proteins were purified with Pierce Protein G Agarose and primary antibody (Flag-tag antibody) in the cold room overnight. Then, the purified proteins were incubated with activated CDK5/p25 and ATP-γ-S. Thiophosphate ester was detected by Western Blotting analysis. **l**, PANC-1 cells were transfected with indicated plasmids. After 24 h, cells were treated with Cycloheximide (CHX) and cells were collected for Western Blotting analysis at different time points. **m**, PANC-1 cells were transfected with indicated plasmids. After 24 h, cells were harvested for Western Blotting analysis. **n**, PANC-1 cells were infected with indicated shRNA. After 72 h, cells were harvested for Western Blotting analysis and RT-qPCR analysis. Data presented as Means ± SD (*n* = 3). N.s., not significant; **, *P* < 0.01; ***, *P* < 0.001. **o**, PANC-1 cells were infected with indicated shRNA. After 72 h, cells were harvested for Western Blotting analysis and RT-qPCR analysis. Data presented as Means ± SD (*n* = 3). N.s., not significant; ***, *P* < 0.001. **p-r**, PANC-1 cells were treated with indicated drugs. Cells were collected for colony formation assay (**p**) and xenografts assay (**q** and **r**). Data presented as Means ± SD (*n* = 3 for O, *n* = 5 for **p** and **q**). ***, *P* < 0.001

Given that PES1 plays a significant role in pancreatic cancer cell proliferation in vivo and in vitro, a specific cell signaling pathway targeting molecules assay was employed to investigate the regulatory mechanism of PES1 in pancreatic cancer cells (Fig. 6a and b). We found that Roscovitine, Dinaciclib, and JQ1 repressed the protein level of PES1 in pancreatic cancer cells (Fig. 6a and b). We have previously reported that JQ1 down-regulated PES1 in liver cancer cells [19]. Here we showed that Dinaciclib decreased PES1 protein levels in a dose-dependent manner (Fig. 6c and d).

Both Roscovitine and Dinaciclib [37] are specific inhibitors of CDK5, and the amino acid sequence of PES1 contains a CDK5 consensus motif (Fig. 6e). Thus, we examined whether CDK5 regulates PES1 in pancreatic cancer. First of all, the endogenous co-immunoprecipitation assay in PANC-1 cells demonstrated that CDK5 interacted with PES1 (EZH2 as internal reference of co-IP assay for CDK5 [20]) (Fig. 5f). Then, the knockdown of CDK5 decreased the protein expression levels of PES1 but not mRNA levels in pancreatic cancer cells (Fig. 6g and h), consistent with the results obtained after Dinaciclib treatment (Fig. 6c and d). Moreover, the inhibition of CDK5 shortened the half-life and increased the ubiquitination level of PES1 in PANC-1 cells (Fig. 6i and j).

To verify further whether CDK5 phosphorylates PES1 and regulates the protein stability of PES1, we constructed mutagenesis plasmids, PES1 S424D and PES1 S424A mutants, to mimic the phosphorylation and de-phosphorylation status of PES1, respectively. We performed the in vitro kinase assay to determine whether PES1 was phosphorylated by CDK5. Wild-type Flag-PES1 and mutant Flag-PES1 (S424A) proteins were translated in vitro. These proteins were purified with Pierce Protein G Agarose and primary antibody (Flag-tag antibody) in a cold room overnight. Then, the purified proteins were incubated with activated CDK5/p25 and ATP-γ-S. A stronger phosphorylation band of PES1 was detected in the Wild-type Flag-PES1 group using a thiophosphate ester antibody than in the mutant Flag-PES1 (S424A) group (Fig. 6k), indicating that CDK5/p25 phosphorylated PES1 on S424 sites. We also demonstrated that PES1 S424D mutants had the most prolonged half-life, but S424A had the shortest half-life in PANC-1 cells (Fig. 6l). Similarly, the phosphorylation status of PES1 showed the least ubiquitination level of PES1 in PANC-1 cells (Fig. 6m). Our data indicate that CDK5 modulated the stability of PES1 in pancreatic cancer cells.

Previous studies have shown that CDK5 increases the expression of c-Myc [38, 39], but the specific mechanism is not known. In this study, we showed that CDK5 stabilized PES1 and PES1 transcriptionally by regulating Myc. We pondered whether CDK5 modulated c-Myc expression via PES1 and found that the knockdown of PES1 diminished the expression of c-Myc in PANC-1 cells (Fig. 6n). On the other hand, the overexpression of CDK5 increased the expression of c-Myc, and this process was blocked by the repression of PES1 (Fig. 6o). Therefore, CDK5/PES1 regulated the expression of c-Myc in PANC-1 cells.

Owing to PES1’s role in cancer cell resistance to JQ1 in pancreatic cancer and Dinaciclib’s ability to destabilize PES1, we next explored the synergistic effect of JQ1 and Dinaciclib in pancreatic cancer cells. Colony formation assay and xenograft assay indicated that Dinaciclib overcame cancer cell resistance to JQ1 in PANC-1 cells in vivo and in vitro (Fig. 6p-6r). These results suggest that CDK5 inhibitors destabilize PES1 and increase cancer cell sensitivity to BET inhibitors in pancreatic cancer cells.

## Discussion

PES1 is a nucleolar protein [18] and is responsible for pre-ribosomal RNA processing [40]. The protein is involved in regulating estrogen-responsive gene transcription by modulating ERα and ERβ balancing [41]. PES1 is also reported to influence telomerase activity and inhibit cellular senescence by interacting with TERT to facilitate telomerase assembly [42]. The nucleolar protein is equally essential for the development of the nervous system and embryogenesis [43, 44]. Highly expressed PES1 is observed in various types of tumors, including breast cancer, gastric cancer, liver cancer, prostate cancer, and neuroblastoma [18]. Abnormally expressed PES1 is associated with poor prognosis in these cancers. Encouragingly, the knockdown of PES1 impedes cancer cell growth and apoptosis. Here, we investigated the clinical characteristic and biological role of PES1 in pancreatic cancer, and our data revealed that PES1 could be a prognostic biomarker of pancreatic cancer, as it enhanced tumor growth in vivo and in vitro, suggesting that PES1 might be a candidate for molecular target therapy of pancreatic cancer.

c-Myc acts as a transcriptional factor that up-regulates or down-regulates the expression of several genes [45]. c-Myc is one of the most important drivers and effectors in promoting the carcinogenesis of pancreatic cancer via the modulation of cell metabolism, cellular growth, metastasis, and apoptosis [46–48] and understanding the regulatory mechanism of c-Myc highlights novel therapeutic strategies for pancreatic cancer. c-Myc is reported to be controlled by many transcriptional factors, including JunD [49], BRD4 [50], and APC [51]. The abnormal activation of oncogenic pathways, such as the PI3K/AKT pathway [52] or MAPK pathway [53], increases c-Myc levels in cancer cells. Here, we revealed that PES1 up-regulated the mRNA and protein levels of c-Myc in pancreatic cancer. We also demonstrated that PES1 bound to BRD4 to increase c-Myc expression in pancreatic cancer cells. Consistent with our findings, Michael Hölzel et al. reported that c-Myc up-regulated the expression of pes1, bop1, and wdr12 involved in ribosome biogenesis and cell metabolism [36]. Consequently, there might be a positive feedback regulation pathway between PES1 and c-Myc in cells.

Epigenetic modifications, including histone methylation [54], histone acetylation [55], and chromatin remodeling [56], are the reversible processes that could regulate gene expression in the cell. The abnormal epigenetic landscape of cancer cells is one of the major reasons for cancer initiation and progression [57]. Importantly, chromatin-modifying enzymes function as mediators during these processes [58]. Inhibiting or improving the activity of chromatin-modifying enzymes has been proven to repress tumorigenesis in pancreatic cancer [57]. Previous reports indicate that BRD4 binds to histone in an acetylation-dependent reaction to modulate gene expression, such as Myc [49].

BET inhibitors competitively interact with the bromodomain of BRD4 to displace the oncogenic protein fused with BRD and inhibit tumor cell growth [11, 59]. The anti-tumor effect of BET inhibitors makes it a potential drug for cancer therapy [60]. However, cancer cell resistance to BET inhibitors hinders their clinical usage. Multiple factors contribute to cancer cell resistance to BET inhibitors in cancer cells. Increased Wnt/β-catenin signaling reportedly resulted in the resistance to BET inhibitors in leukemia [31]. AR can also bind to the bromodomain of BRD4 to decrease the sensitivity of cancer cells to BET inhibitors in prostate cancer [33]. Besides, the protein level of BRD4 has a significant effect on BET inhibitors, and this process is regulated by the activity of SPOP, which degrades BRD4 in prostate cancer cells [30, 32].

In this study, we demonstrated that PES1 inhibition increases the sensitivity of pancreatic cancer cells to BET inhibitors (JQ1). We have previously shown that BRD4 transcriptionally regulated PES1 and JQ1 treatment inhibited PES1 expression [19]. Here we showed further that treatment of the PES1 knock-down group with JQ1 (1 uM) decreased PES1 and c-Myc protein levels more than JQ1 treatment alone or PES1 knock-down alone managed in PANC-1 cells (Additional file 1 Figure S1d and 1e) Remarkably, c-Myc expression level is recognized as the key mediator for BET inhibitors in pancreatic cancer [34]. Therefore, compared with JQ1 treatment alone, the knockdown of PES1 plus JQ1 treatment decreases cancer cell resistance to JQ1 in PANC-1 cells. Additionally, we found that PES1 transcriptionally increased the expression of c-Myc in pancreatic cancer cells through interaction with BRD4, and PES1 might function as an activator to enhance BRD4 activity in pancreatic cancer cells.

Remarkably, we revealed that PES1 was phosphorylated and stabilized by CDK5 in pancreatic cancer cells. Dinaciclib, a CDK5 inhibitor, is in a preclinical trial for the treatment of multiple types of cancer [61], including liver cancer [62], thyroid cancer [63], and chronic lymphocytic leukemia [64]. Dinaciclib can enhance the response to Sorafenib in liver cancer [62]. Other groups have demonstrated that Dinaciclib results in immunogenic cell death and improves the anti-tumor effect of PD-1 antibodies [65]. Moreover, Dinaciclib has been shown to inhibit pancreatic cancer proliferation [21]. Combining Dinaciclib with AKT inhibitors (MK2206) manifests a profound anti-cancer effect in pancreatic patient-derived xenograft models [66]. In this study, our data suggest that Dinaciclib could destabilize PES1 and overcome cancer cell resistance to JQ1 in vivo and in vitro, which provides a therapeutic strategy for the treatment of pancreatic cancer cells.

## Conclusion

**Fig. 7:**
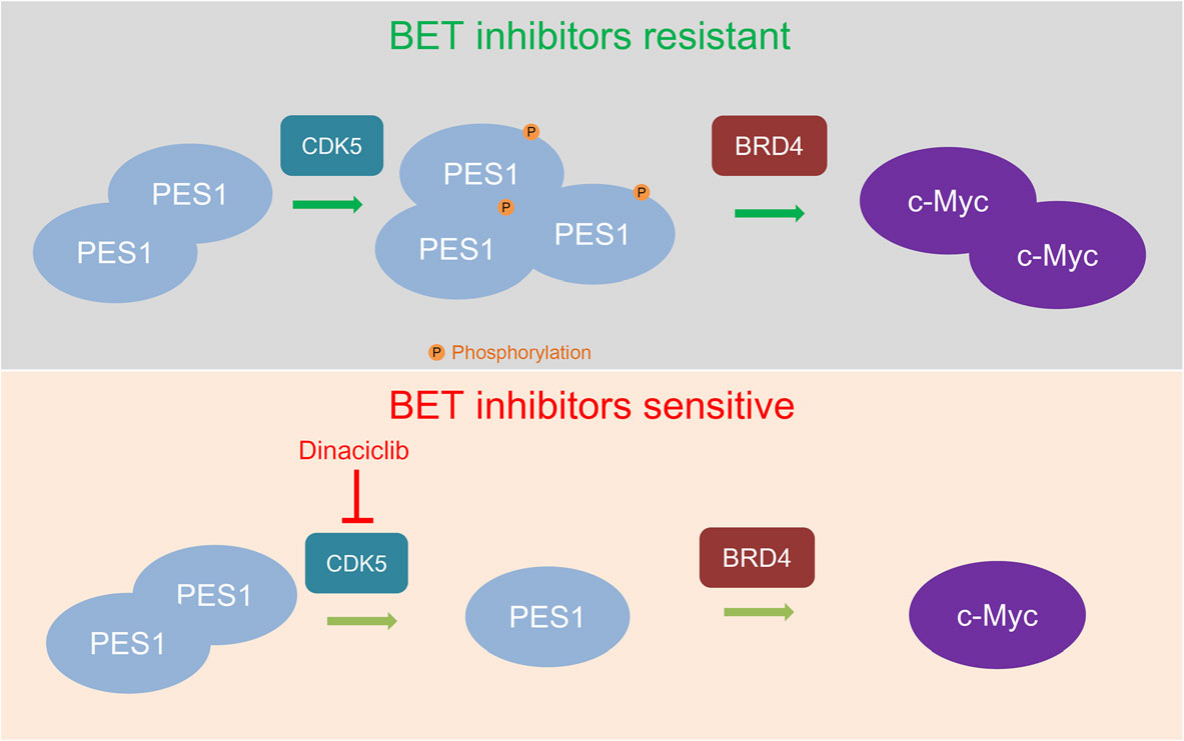
Model depicting that PES1 interacted with BRD4 and contributed to the BET inhibitor resistance via increasing the expression levels of c-Myc in pancreatic cancer. CDK5 phosphorylated and stabilized PES1. Dinaciclib was proved to down-regulate PES1 and overcome the JQ1 resistance in pancreatic cancer cells

Our research has demonstrated that overexpressed PES1 could be considered a prognostic biomarker for pancreatic cancer patients; PES1 promoted pancreatic cancer growth in vivo and in vitro. We also revealed that PES1 interacted with BRD4 and contributed to cancer cell resistance to BET inhibitors by increasing the expression levels of c-Myc in pancreatic cancer. Finally, CDK5 phosphorylated and stabilized PES1, and Dinaciclib was proven to down-regulate PES1 and overcome pancreatic cancer cell resistance to JQ1 (Fig. 7). In conclusion, we not only showed that PES1 had a tumor growth-promoting effect in pancreatic cancer but also demonstrated that combining Dinaciclib with JQ1 could inhibit tumor growth in mouse xenograft models.

## Supplementary information

**Additional file 1: Figure S1.** a and b, PANC-1 cells were transfected with indicated constructs for 48h. Then, these cells were treated with or without JQ1(1 uM) for 24h. Cells were harvested for western blotting (a) and RT-qPCR analysis (b). Data presented as Means ± SD (n = 3). *, P < 0.05; **, P < 0.01; ***, P < 0.001. **c-e**, PANC-1 cells were transfected with indicated constructs for 48h. Cells were harvested for western blotting analysis (c), cell proliferation assay treated with different dose of JQ1 (d) and cells proliferation assay treated with JQ1 (1 uM). Data presented as Means ± SD (n = 4). **, P < 0.01; ***, P < 0.001. **f**, PANC-1 cells were transfected with indicated constructs for 48h. Then, these cells were treated with or without JQ1(10 uM) for 24h. Cells were harvested for RT-qPCR analysis (b). Data presented as Means ± SD (n = 3). ***, P < 0.001. **g**, the whole cell lysate of PANC-1 cell were harvested for western blotting analysis. **Table S1.** Sequences of gene-specific shRNAs. **Table S2.** Sequences of RT-qPCR primers. **Table S3.** Sequences of ChIP-qPCR primers.

## Data Availability

Please contact the corresponding author (Xin Jin, jinxinunion@hust.edu.cn) for data requests.

## Abbreviations

BRCA1: breast cancer associated gene 1
BRD4: Bromodomain-containing protein 4
CDK5: Cyclin dependent kinase 5
CHX: Cycloheximide
IHC: immunohistochemistry
IP: immunoprecipitation
MTS: 3-(4,5-dimethylthiazol-2-yl)-5-(3-carboxymethoxyphenyl)-2-(4-sulfophenyl)-2H-tetrazolium, inner salt
PES1: Pescadillo ribosomal biogenesis factor 1
PMSF: Phenylmethanesulfonyl fluoride
shRNA: short hairpin RNA
TMA: tissue microarray
TSA: Trichostatin A
