# Supplementary material for: PES1 promotes BET inhibitors resistance and cells proliferation through increasing c-Myc expression in pancreatic cancer
Source: J Exp Clin Cancer Res. 2019 Nov 12;38:463. doi: 10.1186/s13046-019-1466-7 (PMC6852745; doi:10.1186/s13046-019-1466-7)
Supplement: Supplementary file 1 — Additional file 1: Figure S1. a and b, PANC-1 cells were transfected with indicated constructs for 48h. Then, these cells were treated with or without JQ1(1 uM) for 24h. Cells were harvested for western blotting (a) and RT-qPCR analysis (b). Data presented as Means ± SD (n = 3). *, P < 0.05; **, P < 0.01; ***, P < 0.001. c-e, PANC-1 cells were transfected with indicated constructs for 48h. Cells were harvested for western blotting analysis (c), cell proliferation assay treated with different dose of JQ1 (d) and cells proliferation assay treated with JQ1 (1 uM). Data presented as Means ± SD (n = 4). **, P < 0.01; ***, P < 0.001. f, PANC-1 cells were transfected with indicated constructs for 48h. Then, these cells were treated with or without JQ1(10 uM) for 24h. Cells were harvested for RT-qPCR analysis (b). Data presented as Means ± SD (n = 3). ***, P < 0.001. g, the whole cell lysate of PANC-1 cell were harvested for western blotting analysis. Table S1. Sequences of gene-specific shRNAs. Table S2. Sequences of RT-qPCR primers. Table S3. Sequences of ChIP-qPCR primers. [file 13046_2019_1466_MOESM1_ESM.docx]

**PES1 promotes BET inhibitors resistance and cells proliferation through increasing c-Myc expression in pancreatic cancer**

Xin Jin, Rui Fang, Ping Fan, Lipeng Zeng, Bin Zhang, Xiaoming Lu, Tao Liu


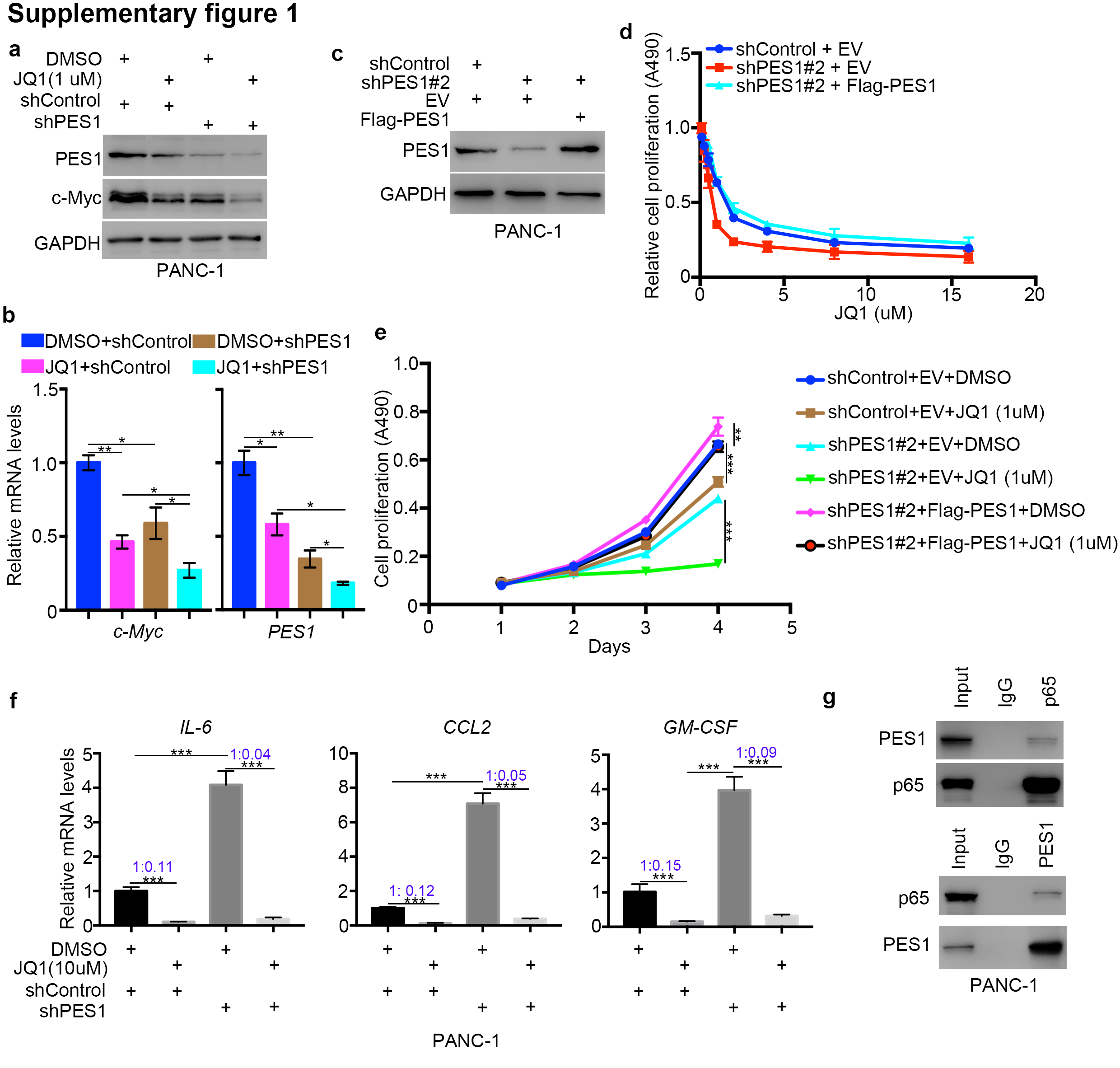


**Supplementary figure 1. a and b,** PANC-1 cells were transfected with indicated constructs for 48h. Then, these cells were treated with or without JQ1(1 uM) for 24h. Cells were harvested for western blotting (a) and RT-qPCR analysis (b). Data presented as Means ± SD (n = 3). *, P < 0.05; **, P < 0.01; ***, P < 0.001. **c-e**, PANC-1 cells were transfected with indicated constructs for 48h. Cells were harvested for western blotting analysis (c), cell proliferation assay treated with different dose of JQ1 (d) and cells proliferation assay treated with JQ1 (1 uM). Data presented as Means ± SD (n = 4). **, P < 0.01; ***, P < 0.001. **f**, PANC-1 cells were transfected with indicated constructs for 48h. Then, these cells were treated with or without JQ1(10 uM) for 24h. Cells were harvested for RT-qPCR analysis (b). Data presented as Means ± SD (n = 3). ***, P < 0.001. **g**, the whole cell lysate of PANC-1 cell were harvested for western blotting analysis.

**Methods and Material**

**Cell lines, cell culture and transfection**

Pancreatic cancer cell lines (HPDE6-C7， AsPC-1， SW1990，MIA PaCa-2，PANC-1， BxPC-3) were purchased from the Chinese Academy of Science Cell Bank. All cell lines were cultured in Dulbecco's modified Eagle’s medium (DMEM) medium (Invitrogen, USA) containing 10% fetal bovine serum (FBS) (HyClone, USA). All cell lines were kept in a 37°C incubator at 5% CO_2_. PANC-1 and BxPC-3 cells were seeded in 6-well plates at a density of 2.5×10^5^ cells per well for 24 hours before transfection. Transfections were performed by using Lipofectamine 2000 (Thermo Fisher Scientific). The lentivirus-based control and gene-specific shRNAs (shCDK5#1, shCDK5#2, shBRD4#1, shBRD4#2, Sigma-Aldrich) were employed to interfere the expression of gene in 293T cells. After transfecting lasts 24 hours, the transfection medium was substitute for prepared DMEM, containing 10% FBS and 1mM sodium pyruvate. Then the virus culture solution was gathered after 48 hours of continuous culture and mixed into PANC-1 and BxPC-3 cells lines supplemented with 12 μg/ml of polybrene. 10μg/ml of puromycin was used to filtrate successfully infected cells after mixing for 24 hours. The specific sequence information of shRNAs was provided in supplementary Table S1.

**Real-time RT-PCR**

Trizol reagent (Thermo Fisher Scientific, USA) was used to extract total RNA in PANC-1 and BxPC-3 cells. The we conducted reverse transcription to make total RNA translate persistent cDNA on the basis of specification (PrimeScript™ RT reagent Kit). Finally, RT-PCR analysis was carried out to amplificate the cDNA according to a PCR kit (TB Green™ Fast qPCR Mix). All the cycle index was normalized to GAPDH, and the 2-ΔCq method was used to describe the multiple variation. The forward and reverse primer sequences of PES1, c-Myc and GAPDH were provided in Supplementary Table S2.

**Chromatin immunoprecipitation (ChIP) and ChIP-qPCR**

ChIP was performed following the manufacturer’s instructions with the Chromatin Extraction Kit (Abcam, ab117152, USA) and ChIP Kit Magnetic - One Step (Abcam, ab156907, USA) (Fan et al., 2018a). BRD4 (Cell signaling Technology, 13440, dilution 1:50) was used for the ChIP assay. The purified DNA was analyzed by real-time PCR with a PCR kit (Takara Bio Inc., Japan) according to the manufacturer’s protocols (Jin et al., 2018). The specific sequence information of shRNAs was provided in supplementary Table S3.

**Survival analysis and correlation analysis using GEPIA web tool**

The online database Gene Expression Profiling Interactive Analysis (GEPIA, http://gepia.cancerpku.cn/index.html) was used to analyze RNA sequencing expression data related to our project based on The Cancer Genome Atlas (TCGA) and the Genotype-Tissue Expression (GTEx) projects. GEPIA performs survival analyses based on gene expression levels and uses a log-rank test for hypothesis evaluation. GEPIA also performs a pairwise gene correlation analysis for any given sets of TCGA and/or GTEx expression data using Pearson correlation statistics.

**Supplementary Table S1: Sequences of gene-specific shRNAs**

| shPES1-1 | 5′- CGAGAGTTGTATGGAGAAA -3′ |
| --- | --- |
| shPES1-2 | 5′- GCATCACCCATCAGATTGT -3′ |
| shBRD4-1 | 5′- GTACCGGTGAACCTCCCTGATTACTATACTCGAGTATAGTAATCAGGGAGGTTCATTTG-3′ |
| shBRD4-2 | 5′- CCGGCCTGGAGATGACATAGTCTTACTCGAGTAAGACTATGTCATCTCCAGGTTTTTG-3′ |
| shCDK5-1 | 5′- CCGGCCTGAGATTGTAAAGTCATTCCTCGAGGAATGACTTTACAATCTCAGGTTTTTTG-3′ |
| shCDK5-2 | 5′- CCGGCAGAACCTTCTGAAGTGTAACCTCGAGGTTACACTTCAGAAGGTTCTGTTTTTTG-3′ |

**Supplementary Table S2: Sequences of RT-qPCR primers**

| **Species** | **Gene** | **Forward (5’-3’)** | **Reverse (5’-3’)** |
| --- | --- | --- | --- |
| Human | *GAPDH* | ACCCAGAAGACTGTGGATGG | TTCAGCTCAGGGATGACCTT |
| Human | *PES1* | GAGGGCCTGAAGTTCTTCCT | CAATCTGATGGGTGATGCGG |
| Human | *Myc* | TTCGGGTAGTGGAAAACCAG | CAGCAGCTCGAATTTCTTCC |
| Human | *IL-6* | AGTCCTGATCCAGTTCCTGC | CTACATTTGCCGAAGAGCCC |
| Human | *CCL2* | CAATCAATGCCCCAGTCACC | TCGGAGTTTGGGTTTGCTTG |
| Human | *GM-CSF (CSF2)* | GCGTCTCCTGAACCTGAGTA | TGGGTTGCACAGGAAGTTTC |

**Supplementary Table S3: Sequences of ChIP-qPCR primers**

| **Species** | **Gene** | **Forward (5’-3’)** | **Reverse (5’-3’)** |
| --- | --- | --- | --- |
| Human | *Myc* | CAGCGGTCCGCAACCCTTGCC | CGCGTCGGGAGAGTCGCGTCC |
